# Analysis on Node Position of Imperfect Resonators for Cylindrical Shell Gyroscopes

**DOI:** 10.3390/s16081206

**Published:** 2016-07-30

**Authors:** Zidan Wang, Yulie Wu, Xiang Xi, Yongmeng Zhang, Xuezhong Wu

**Affiliations:** College of Mechatronics Engineering and Automation, National University of Defense Technology, Changsha 410073, China; wzdnudt@163.com (Z.W.); fordada@126.com (X.X.); zymnudt@163.com (Y.Z.)

**Keywords:** node position, imperfect resonator, trimming method

## Abstract

For cylindrical shell gyroscopes, node position of their operating eigenmodes has an important influence on the gyroscopes’ performance. It is considered that the nodes are equally separated from each other by 90° when the resonator vibrates in the standing wave eigenmode. However, we found that, due to manufacturing errors and trimming, the nodes may not be equally distributed. This paper mainly analyzes the influences of unbalanced masses on the cylindrical resonators’ node position, by using FEM simulation and experimental measurement.

## 1. Introduction

As a kind of solid-state wave gyroscope, the cylindrical shell vibrating gyroscope can be used to measure the angular velocity of a rotating body based on the inertia effect of the standing wave in two vibration modes of the axial symmetric resonator [1], which have advantages such as small size, high operation accuracy, low cost, low power consumption, good shock resistance, and long life [2]. The advantages offered by the cylindrical shell vibrating gyroscope have always been of great interest to many researchers, institutes and companies.

The Innalabs Holding’s Coriolis Vibration Gyroscope (CVG) and the Watson Industries’ Vibrating Structure Gyroscope (VSG) are typical cylinder shell vibrating gyroscopes [3,4]. Besides, Marconi Avionics has developed the START vibrating gyroscope [5].

The working cylindrical resonator vibrating in its second-order mode has a maximum vibration amplitude at four antinodes and a zero vibration amplitude at four nodes. For a perfect resonator, the nodes are equally distributed 90° from each other [6]. However, various kinds of cylindrical shell gyroscopes with different structures will have inhomogeneous errors after manufacturing, leading to the instability of vibratory modes and node positions [7]. Experts home and abroad have studied different kinds of inhomogeneous errors and the resultant quality imperfection.

Fox at the University of Nottingham researched the vibratory theory and mode trimming principles of ring and cylindrical gyroscopes and proved that by adjusting part masses and rigidity, the frequency split can be reduced [8,9]. Zhbanov revealed the influence of the movability of the resonator center on the operation of the hemispherical gyroscope [10]. Dag Kristiansen studied the nonlinear oscillations and the problem of superharmonic resonances [11]. Dzhashitov in Russia conducted a theoretical analysis on the model of thermo-elastic stress-strain states and revealed the inner relationship between temperature and stress states [12].

At present, papers researching the characteristics of nodes in cylindrical resonators are quite few, in spite of those studies on the errors of imperfect resonators. In fact, it is necessary to study the characteristics of nodes, for they are closely related to the zero-bias of the gyroscope. This paper will develop an in-depth study in this field, revealing how mass, rigidity, and geometric errors affect the localization of nodes, and providing helpful instructions for manufacture, trimming and accuracy control of high quality resonators.

## 2. Structure and Working Principle

**Figure 1:**
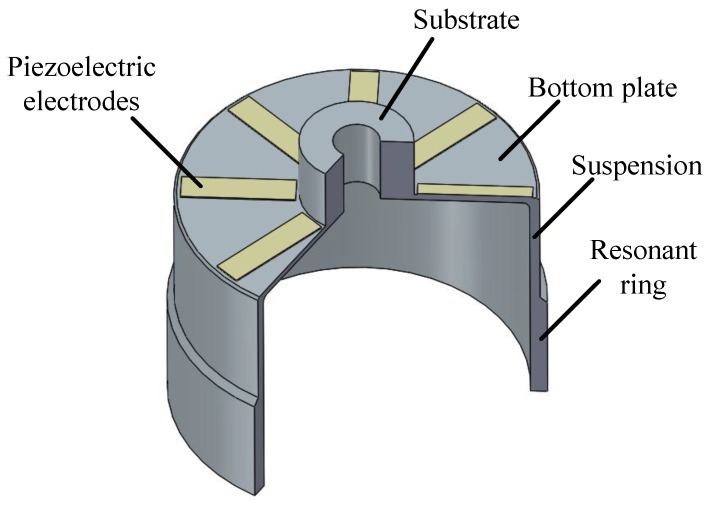
Structure of the cylindrical resonator.

A typical resonant shell of the cylindrical gyro is shown in Figure 1. The structure comprises a rigid substrate, a round bottom, a cylindrical suspension and a thick resonant ring. Eight piezoelectric electrodes are glued on the bottom. The vibration of the cylindrical wall is excited and then converted to a voltage signal via the electrodes.

**Figure 2:**
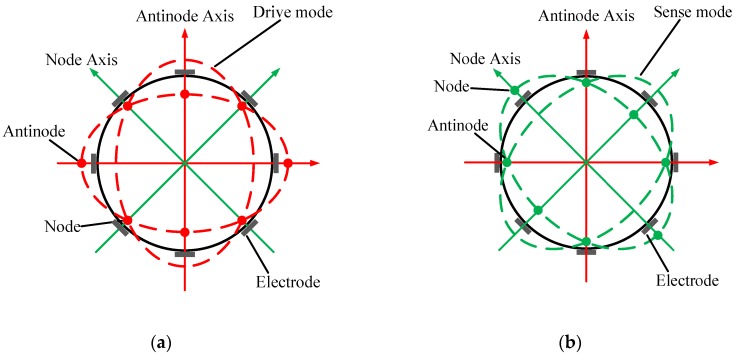
Mode shape of a perfect resonator. (**a**) Drive mode; (**b**) Sense mode.

The working principle of the resonator is well understood. Figure 2 describes the vibration pattern of a perfect resonator. There is a pair of operating modes known as the drive mode and sense mode, which are separated from each other by 45°. The operating modes have the same natural frequency and the mode shape in the form of a standing wave has four antinodes and four nodes. The drive mode of the shell is used to generate the standing vibration along the exciting orientation. When the gyroscope rotates, the sense mode can be detected by the electrodes due to the Coriolis effect.

## 3. ANSYS Simulation on Node Position

According to the preceding description of a perfect resonator, the antinodes and nodes are 45° apart from each other. However, the mode shape of an imperfect resonator is related to the complex theory of shell dynamics and numerical solutions are difficult to obtain. As a result, the FEM software ANSYS is employed to analyze the node localization of imperfect resonators. It is worth mentioning that FEM can induce an artificial frequency split of 0.2 Hz to a perfect resonator with a general mesh size used in our research. However, this paper mainly discusses the conditions with large frequency splits caused by distinct geometric errors. So the artificial frequency split of a perfect resonator is small enough to be neglected.

### 3.1. Finite Element Modeling of the Resonator

**Figure 3:**
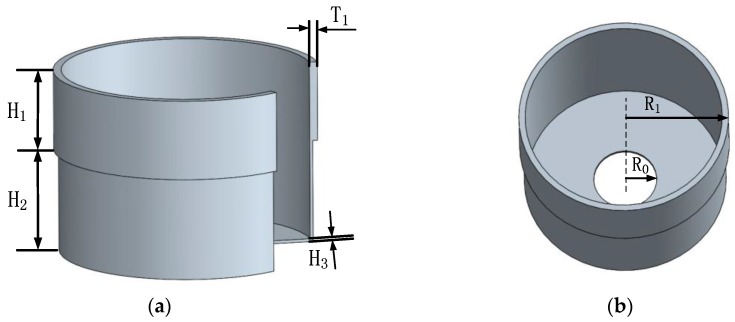
The geometric model of the resonator. (**a**) The partial sectional model; (**b**) The entire geometric model.

The geometry parameters listed in Table 1 are used to build the model (shown in Figure 3), and the material properties are listed in Table 2. Further, the element type SOLID186 is chosen. Then, different kinds of imperfections are exerted on the FE model in order to reveal their influence on the operating eigenmodes and the node position. By means of modal analysis, the displacement and the azimuth of every point on a circle with an interval of 1° are extracted. The position with minimum displacement is considered to be where the nodes are located.

### 3.2. Influence of Mass Variation on Node Position

The mass variation relative to a perfect resonator is a common imperfection. The mass distribution may not be homogeneous after machining because of the limited machining accuracy or the inhomogeneity of the material density. To simplify this condition, rectangular mass is added on the original model before simulative modal analysis.

#### 3.2.1. Influence of Single Added Mass

**Figure 4:**
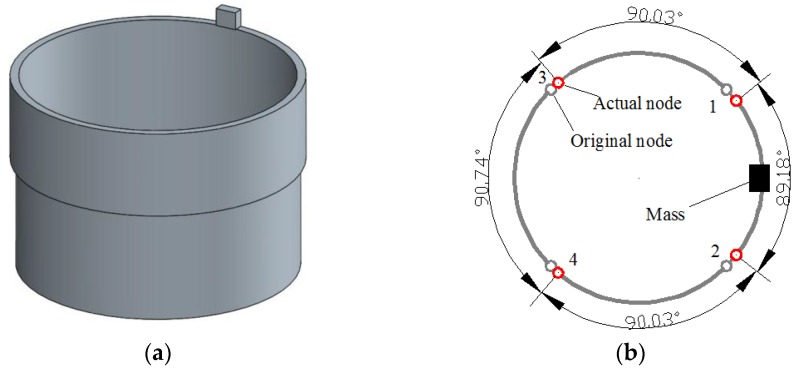
Resonator condition with single added mass. (**a**) The geometric model applied in simulation; (**b**) The node position in this case.

As is shown in Figure 4, when a 2.8 mm × 2.2 mm × 1 mm rectangular mass is added on the top of the resonator ring (not on the internal or external surface to ensure that it does not increase the radial rigidity too much), the frequency split reaches 30 Hz. Furthermore, the four nodes are all shifting towards the added mass.

#### 3.2.2. Influence of Double Added Mass

**Figure 5:**
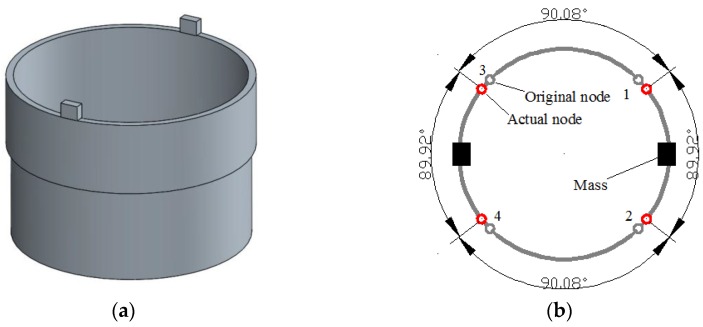
Resonator condition with two added masses. (**a**) The geometric model applied in simulation; (**b**) The node position in this case.

**Figure 6:**
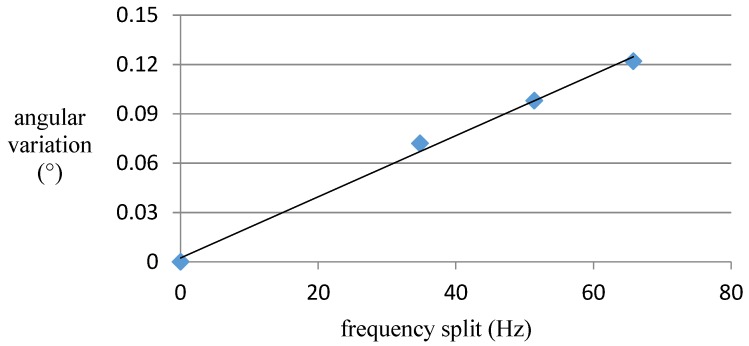
The relationship between angular variation and frequency split.

In this case, which is shown in Figure 5, when two rectangular masses are added on the resonator ring, the actual node position has moved slightly away from the ideal position. However, even though the frequency split caused by the mass has reached 60 Hz, the angular error of the nodes remains within merely 0.12° (shown in Figure 6 and Table 3). Since the frequency split of our resonators can be controlled within 5 Hz after precision manufacture, a 60 Hz frequency split represents quite a large mass disturbance. In summary, we can conclude that simple mass disturbance has a limited influence on the node position.

### 3.3. Influence of Rigidity Variation on Node Position

Trimming is a key process to eliminate manufacture error and frequency split. It is considered that trimming a tiny quantity of material off the resonator will lead to the obvious variation of the natural frequency and mode shape [13] as a result of the rigidity change in this process.

**Figure 7:**
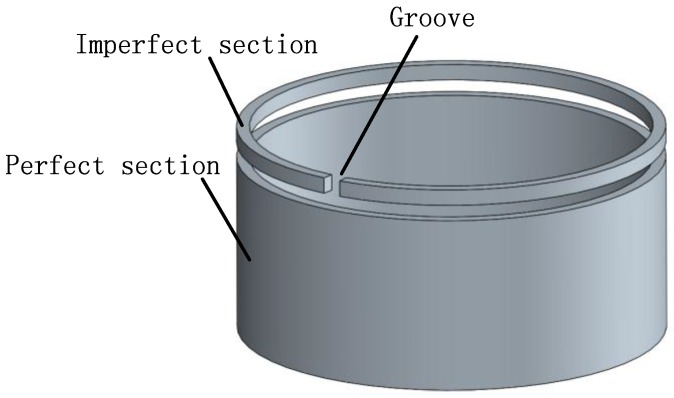
The rigidity condition.

Firstly, the rigidity condition of the trimmed resonator is illustrated (shown in Figure 7). Suppose that a resonator is trimmed and some of the resonator rings are ‘cut’, and the radial rigidity will vary as the trimming groove position changes.

**Figure 8:**
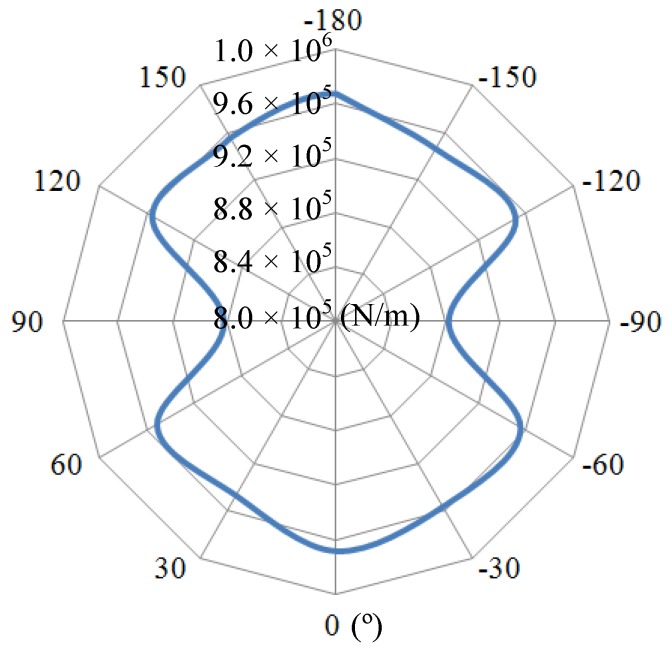
The rigidity condition of a trimmed resonator.

Afterwards, the calculation presents that a 2.2 mm × 1.2 mm trimming groove causes a rigidity variation by 8.8%, while its mass has merely changed by 0.26%, which reveals that trimming grooves cause a larger variation in rigidity than in mass (shown in Figure 8).

#### 3.3.1. Influence of Single Trimming Groove on Node Position

**Figure 9:**
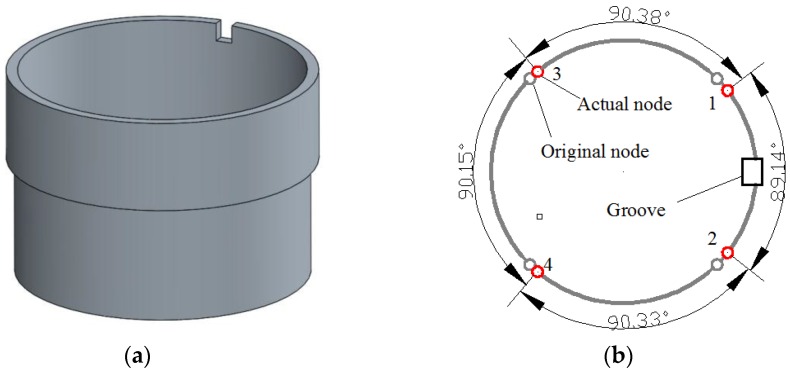
Resonator condition with one groove in simulation. (**a**) The geometric model applied in simulation; (**b**) The node position in this case.

**Figure 10:**
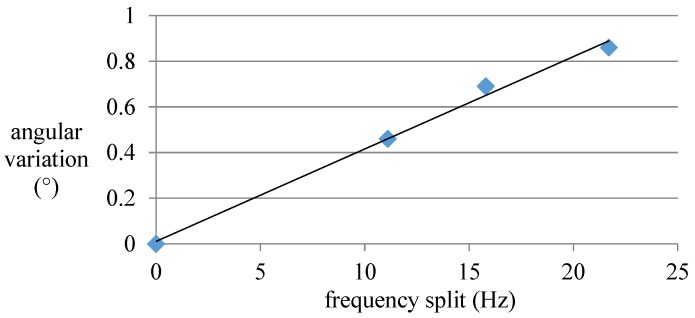
The relationship between angular variation and frequency split.

From Figure 9, since the trimming groove lacks position constraint, the actual nodes are ′pulled′ to the groove, resulting in the relative position change of nodes in the same side. Also, the angular variation is proportional to the groove depth. It can reach 0.86° with a 2.2 mm deep× 1.2 mm wide trimming groove. In addition, in view of the considerable angular variation values in Figure 10, we can come to a conclusion that groove structure has a great influence on the node position.

#### 3.3.2. Influence of Multiple Trimming Groove on Node Position

In the interest of a deeper-level exploration on the effect of the groove structure, the conditions of multiple trimming grooves are considered below.

**Figure 11:**
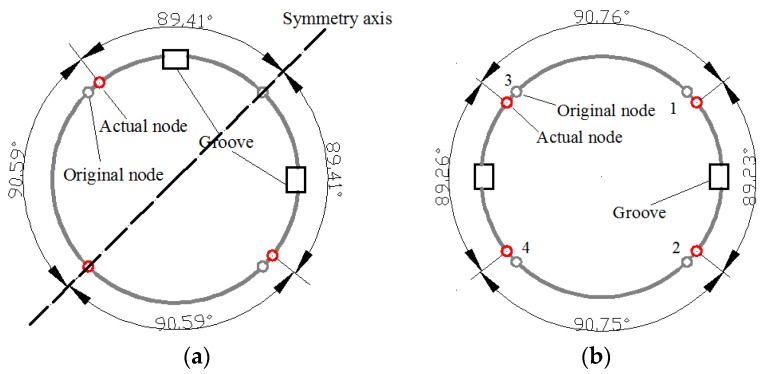
Simulation results with two grooves. (**a**) The node position with two grooves at a 90° angle; (**b**) The node position with two grooves at a 180° angle.

Firstly, two 2.2 mm deep × 1.2 mm wide trimming grooves are applied on the resonator ring at a 90° angle. The consequent mode pattern is shown in Figure 11a, where the two nodes on the symmetry axis remain unchanged while two side nodes shift towards the groove. The variable trend of the nodes is similar to the former condition with one groove.

Similarly, when two 2.2-mm-deep trimming grooves are applied on the resonator ring at a 180° angle, the angular variation becomes 0.77°, which is a rather big angle (shown in Figure 11b).

However, when four evenly distributed trimming grooves are applied on the resonator ring, the angular variation is merely 0.01°, even though the groove depth is increased to 1.5 mm. The angular variation is usually considered to be zero in this condition. However, since the depths of the grooves are not exactly the same, different trimmed resonators actually have a distinct node position.

### 3.4. Influence of Roundness Error on Node Position

Roundness (the difference between the maximum outer diameter and the minimum outer diameter) is a significant property for evaluating the manufacture accuracy of the resonator.

**Figure 12:**
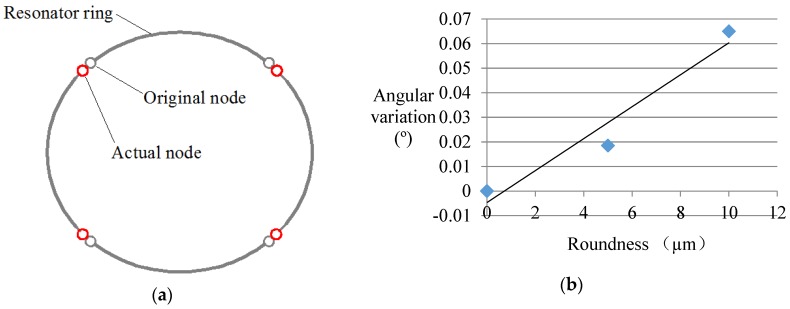
Simulation condition with roundness error. (**a**) The node position in this case; (**b**) The relationship between angular variation and roundness.

Apart from the groove structures, the shape of the resonator ring will also cause node position variation. In this case, the distribution of the nodes depends on the specific shape of the resonator. An example of an elliptical resonator is given out in this paper. As is shown in Figure 12, the nodes of the resonator have a trend of approaching the long axis. The larger the ellipticity (the deviation from the perfect circular form to the elliptic form) is, the greater the angular variation becomes. In addition, 10 mm roundness leads to about a 0.06° angular error. Considering that precision machining on the resonator shall be guaranteed at the micron scale, the node error caused by shape error is rather small.

In summary, in view of our machining condition, the node position variation is more due to trimming grooves than roundness error.

### 3.5. Analysis on Rigidity Variation and Angular Error Caused by Digging Trimming

**Figure 13:**
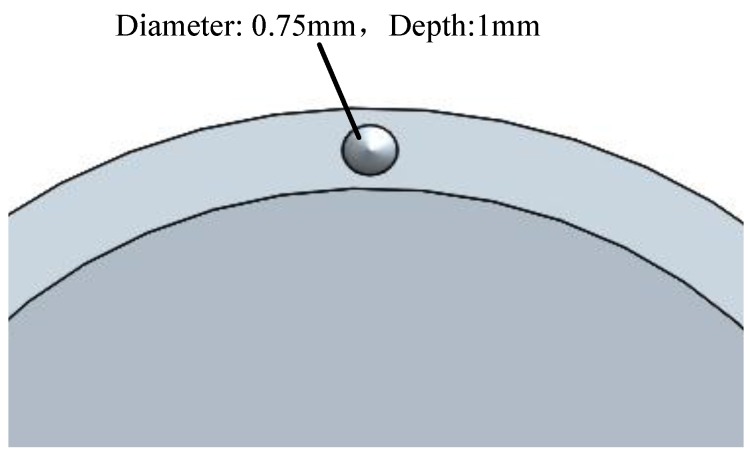
Influence of roundness error.

Removing mass near the mid-line of the resonator ring leads to a limited change of the radial rigidity. With the removing method shown in Figure 13, the rigidity merely varies by 0.18%, while the rigidity change caused by trimming grooves can reach 2%.

Digging trimming is a kind of mass trimming. It causes less node position error. A single dug pit can eliminate a 3 Hz frequency split, bringing a 0.01° angular variation.

## 4. Experiment

In order to verify the simulation results, acoustic experiments are implemented to detect the node position in specific imperfection cases.

### 4.1. Test Equipments and System

**Figure 14:**
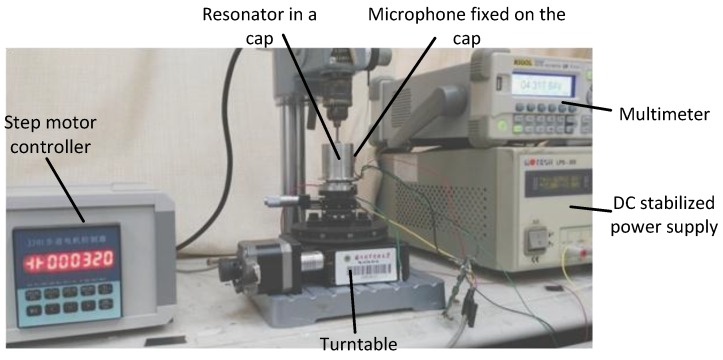
Experiment system.

An acoustic test system is set up to carry out the experiments, and it is shown in Figure 14. In order to acquire the accurate angular position of the four nodes, the output signals in circumferential positions should be measured. Firstly, piezoelectrodes are used to drive the resonator fixed on the turntable and the driving frequency is stabilized at the eigenfrequency of the drive mode. Secondly, the acoustic signals are detected by a microphone at places every 0.2°, while the location with minimal output can be considered as a node. We can change the detecting position by rotating the turntable controlled by the step motor controller. At last, the angle between nodes can be counted from the step motor controller screen.

### 4.2. Influence of Single Groove on Node Position

**Figure 15:**
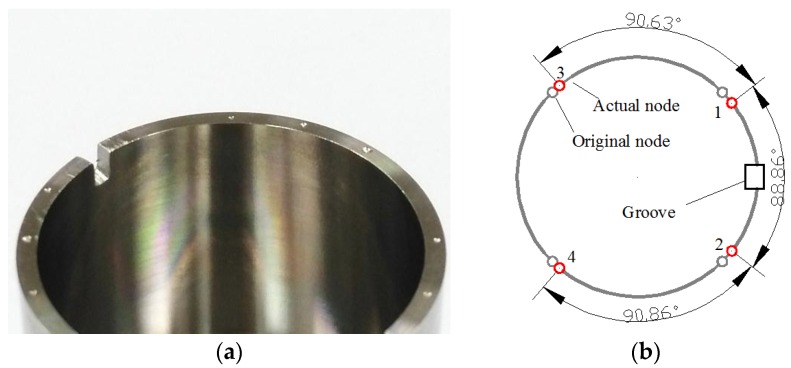
Resonator condition with one groove in experiment. (**a**) The tested resonator; (**b**) The experiment result of node position.

Figure 15 describes the experimental results for this condition. As a 1.2-mm-deep groove is cut out, the angle between node 1 and node 2 near the groove is about 88.86°, much smaller than 90°. Meanwhile, the two angles between node 1 and node 3, and node 2 and node 4 become obviously larger than before, reaching 90.63° and 90.86°, respectively.

Furthermore, when the groove is trimmed to 1.8 mm deep, the angle between node 1 and node 2 becomes as small as 88.45°, and the angles on the two sides are 90.84° and 91.33°. Thus, we can see that the deeper the groove is, the larger the angular variation becomes.

### 4.3. Influence of Two Grooves on Node Position

**Figure 16:**
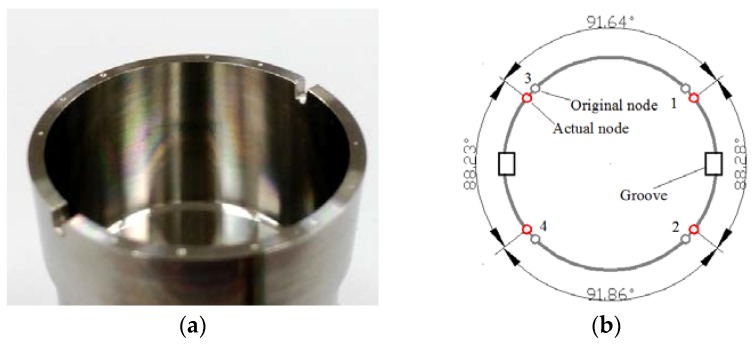
Resonator condition with two grooves in experiment. (**a**) The tested resonator; (**b**) The experiment result of node position.

Two 1.2-mm-wide and 1.8-mm-deep rectangular grooves are cut out at 0° and 180° (shown in Figure 16a). From the statistic data we can see that the angle between node 1 and node 2 is 88.28° while the opposite angle between node 3 and node 4 is 88.23° (shown in Figure 16b).

### 4.4. Influence of Added Mass on Node Position

**Figure 17:**
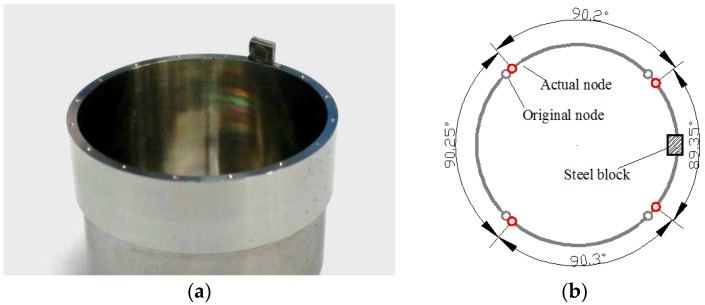
Resonator condition with one added mass in experiment. (**a**) The tested resonator; (**b**) The experiment result of node position.

When a 2.8 mm × 2.2 mm × 1 mm small mass is glued on the top of the resonator edge (shown in Figure 17a), all four nodes are moved towards the position of the mass (shown in Figure 17b). In addition, when a larger mass is added, the shift of the nodes becomes more apparent. Therefore, the experiment results can well fit the simulation.

## 5. Conclusions

In this paper, the influence principle of different errors on node position is studied. Simulation results show that mass disturbance and roundness error exert little influence on the node position while rigidity variation caused by trimming grooves exerts a significant influence on the node position. Then vibration experiments on resonators with specific imperfections are implemented to validate the mentioned situations, showing that simulated and measured results are in close agreement.

Therefore, in order to avoid rigidity failure, mass trimming is recommended. Considering that the node position is relative to the zero bias stability, the present work is useful for performance improvement of the resonator.

## Figures and Tables

**Table 1:** Material properties of the cylindrical resonator.

Parameter	Value
Young′s modulus E	210 GPa
Poisson′s ratio μ	0.3
Density ρ	7800 kg/m^3^

**Table 2:** Structure parameters of the resonator.

Parameter	Value
Height of resonant ring H_1_	8 mm
Height of suspension ring H_2_	10 mm
Radius of substrate R_0_	4 mm
Internal diameter of resonator R_1_	12 mm
Thickness of resonant ring T_1_	1 mm
Thickness of suspension ring T_2_	0.3 mm
Thickness of bottom H_3_	0.3 mm

**Table 3:** Frequency splits and node position variations due to mass disturbance.

Side Length of a 1 mm Thick Square Block (mm)	Volume (mm^3^)	Frequency Split (Hz)	Node Position Variation (°)
0	0	0	0
1.8	3.24	34.8	0.072
2.2	4.84	51.4	0.098
2.5	6.25	65.8	0.122
